# *GFI1* Is Repressed by p53 and Inhibits DNA Damage-Induced Apoptosis

**DOI:** 10.1371/journal.pone.0073542

**Published:** 2013-09-04

**Authors:** Pei Du, Fangqiang Tang, Yaling Qiu, Fan Dong

**Affiliations:** Department of Biological Sciences, University of Toledo, Toledo, Ohio, United States of America; Roswell Park Cancer Institute, United States of America

## Abstract

GFI1 is a transcriptional repressor that plays a critical role in hematopoiesis and has also been implicated in lymphomagenesis. It is still poorly understood how GFI1 expression is regulated in the hematopoietic system. We show here that *GFI1* transcription was repressed by the tumor suppressor p53 in hematopoietic cells. Knockdown of p53 resulted in increased GFI1 expression and abolished DNA damage-induced GFI1 downregulation. In contrast, GFI1 expression was reduced and its downregulation in response to DNA damage was rescued upon restoration of p53 function in p53-deficient cells. In luciferase reporter assays, wild type p53, but not a DNA binding-defective p53 mutant, repressed the *GFI1* promoter. Chromatin immunoprecipitation (ChIP) assays demonstrated that p53 bound to the proximal region of the *GFI1* promoter. Detailed mapping of the *GFI1* promoter indicated that *GFI1* core promoter region spanning from −33 to +6 bp is sufficient for p53-mediated repression. This core promoter region contains a putative p53 repressive response element, mutation of which abolished p53 binding to and repression of *GFI1* promoter. Significantly, apoptosis induced by DNA damage was inhibited upon Gfi1 overexpression, but augmented following GFI1 knockdown. Our data establish for the first time that *GFI1* is repressed by p53 and add to our understanding of the roles of GFI1 in normal hematopoiesis and lymphomagenesis.

## Introduction

*Growth Factor Independence 1* (*GFI1)* encodes a nuclear zinc-finger (ZF) transcriptional repressor that plays a critical role in hematopoiesis [1], [2]. Loss of *Gfi1* in mice leads to markedly reduced thymic cellularity due to increased apoptosis and reduced proliferation during early T cell development. The numbers of early and mature B cells are also reduced in *Gfi1* ^−/−^ mice. Gfi1 has been shown to promote the terminal granulocytic differentiation and antagonize the alternative development towards macrophages. Myeloid precursors from *Gfi1* ^−/−^ mice are unable to differentiate into mature neutrophils *in vitro*, but instead give rise to atypical monocytes. In addition, Gfi1 has been implicated in regulating hematopoietic stem cell (HSC) self-renewal and survival [3]–[5]. *Gfi1* ^−/−^ HSCs are hyper-proliferative, but show increased sensitivity to stress-induced apoptosis and are impaired in the ability to sustain long-term hematopoiesis.

*Gfi1* was originally identified as a frequent target of proviral insertion in T and B cell lymphomas [6]–[12], which resulted in the activation of *Gfi1* at both the transcriptional and post-transcriptional levels [13], [14]. High levels of Gfi1 in T and B cells abolish G1 cell cycle arrest and apoptosis induced by growth factor withdrawal [15]–[17]. Transgenic mice overexpressing Gfi1 in T cells were predisposed to T cell lymphoma [8], [9], [18]. In contrast, *Gfi1* ablation cured mice from acute lymphoblastic leukemia (ALL) and limited the expansion of primary T-cell ALL xenografts in mice [19]. Furthermore, Gfi1 has been shown to cooperate with a number of oncoproteins including Myc, Pim-1, E2A-HLF and the intracellular form of Notch1 (ICN) in the development of lymphoma and ALL [8], [9], [14], [18], [19]. Thus, Gfi1 functions as an oncoprotein in the lymphoid system. Notably, unlike its role in lymphoid system, Gfi1 appears to function as a tumor suppressor in the myeloid system [1], .

The tumor suppressor p53 is a transcription factor that is activated by cellular stresses such as DNA damage and oncogene stimulation [22], [23]. Upon activation, p53 may induce cell cycle arrest, DNA repair, cellular senescence and apoptosis. Thus, p53 acts to maintain genome stability and eliminate damaged or abnormally proliferating cells. Loss of p53 function is the most common event during tumorigenesis. The biological activities of p53 are mediated mainly through transcriptional regulation of its target genes. p53 has been shown to activate a wide range of target genes including those that exert a negative effect on cell proliferation and survival [24], [25]. In addition to transcriptional activation, p53 also functions to repress certain genes and recent studies indicate that transcriptional repression by p53 is required for its tumor suppressor activity [25], [26]. However, it is still poorly understood about the molecular mechanisms by which p53 represses gene expression.

Here we report for the first time that p53 represses *GFI1* transcription in a manner that is dependent on its DNA binding activity. We also show that a 40-bp core promoter region of *GFI1* was required and sufficient for p53-mediated repression. A close examination of the 40-bp promoter fragment revealed a putative p53 response element (RE), mutation of which blocked p53 binding to and repression of *GFI1*. We further demonstrate that GFI1 functioned to inhibit DNA damage-induced apoptosis. Our data identify *GFI1* as a new p53-repressed target gene and suggest that GFI1 downregulation may be important for the tumor suppressor action of p53 in the hematopoietic system.

## Materials and Methods

### Cells

Murine Pro-B Ba/F3 cells [27] were maintained in RPMI-1640 medium supplemented with 4% fetal bovine serum (FBS), 4% WEHI-3B cell-conditioned media as a crude source of murine interleukin-3, and 1% penicillin/streptomycin (P/S). Human Burkitt lymphoma cell line Ramos (American Type Culture Collection), human T lymphoblastic cell line Molt3 [28], and human myeloid leukemic cell lines U937 [29] and HL-60 [30] were cultured in RPMI-1640 medium supplemented with 10% FBS and 1% P/S solution. Human megakaryocytic cell line MO7e [31] was cultured in RPMI-1640 medium supplemented with 10% FBS and 5 ng/ml recombinant human GM-CSF. Human colon cancer cell line HCT116 and its p53^−/−^ and p21^−/−^ derivatives [32], [33], originally generated by Dr. Bert Vogelstein (Johns Hopkins University), were kindly provided by Dr. William Taylor (University of Toledo) and cultured in Dulbecco’s modified Eagle’s medium (DMEM) supplemented with 10% FBS and 1% P/S solution. The cells were grown in a humidified incubator at 37°C with 5% CO_2_.

### Reagents

Antibodies against Gfi1 (N-20) and human p53 (BP 53.12) were purchased from Santa Cruz Biotechnology. Antibodies against Bax and Bcl2 were purchased from BD Transduction. Anti Bak and anti β-actin antibodies were from Calbiochem and Sigma, respectively. QuikChange™ Site-Directed Mutagenesis Kit was purchased from Stratagene.

### Expression Constructs and Stable Gene Delivery

The pBabePuro.p53ER^tam^ expression construct [34] was a generous gift from Dr. Douglas R. Green (La Jolla Institute for Allergy and Immunology). HL-60 and U937 cells were transfected with pBabePuro.p53ER^tam^ by electroporation and selected in 2 µg/ml puromycin 48 hrs later. Puromycin-resistant cells were subcloned by limiting dilution. Individual clones were examined for expression of p53ER^tam^ by Western blot analysis.

For inducible expression of Gfi1, the Gfi1-RV retroviral vector [17] was digested with *BamHI* and *XbaI*, and the resulting DNA fragment of approximate 2.8 kb, which contained the rat Gfi1 cDNA, an internal ribosomal entry sequences (IRES) and humanized GFP cDNA, was used to replace the mSEAP cDNA of the lentiviral vector pTMPrtTA [35], a generous gift of Dr. Olivier Danos (Genethon-Centre National de la Recherche Scientifique, France). 293T cells were transfected with pPMPrtTA-Gfi1-GFP construct along with psPAX2 and pMD2G using the calcium phosphate coprecipitation method. Supernatants containing the lentivirus were harvested 48 and 72 hrs later. Ba/F3 and Ramos cells were infected with the lentivirus in the presence of 8 µg/ml of polybrene and treated with 1 µg/ml doxycycline (Doxy) 72 hrs post infection. GFP positive cells were sorted and then returned to culture media without Doxy.

### RNA Interference

Lentiviral constructs encoding human GFI1 specific shRNAs have been described before [36],[37]. Lentiviral construct containing human p53 shRNA was a gift of Dr. Robert A. Weinberg (Addegene plasmid #19119). 293T cells were transfected with the lentiviral constructs along with the packaging plasmids using the calcium phosphate coprecipitation procedure. The virus-containing supernatants were harvested and used to infect cells as described above. Cells were selected in 2 µg/ml puromycin 48 h later and shRNA-mediated knockdown of GFI1 and p53 was examined by Western blot analysis.

### Chromatin Immunoprecipitation (ChIP) Assay

ChIP assays were performed essentially as described [36], [37]. Briefly, cells were fixed with 1% formaldehyde and then lysed in hypotonic buffer [5 mM Tris-HCl (pH 7.5), 85 mM KCl and 0.5% Nonidet P-40]. After centrifugation at 6000 rpm for 5 min, nuclei were lysed in ChIP lysis buffer [1% SDS, 10 mM EDTA, and 50 mMTris HCl (pH 7.5)] and sonicated to shear chromatin DNA to ∼500-bp fragments. Nuclear lysates were precleared with protein A/G agarose beads and rabbit normal IgG for 1 h and subjected to immunoprecipitation using the anti p53 or a species-matched irrelevant antibody. Precipitated DNA was examined by semi-quantitative PCR.

### Promoter Constructs and Luciferase Reporter Assay

The human *GFI1* promoter fragments spanning from −1933 bp to +468 bp and from −4840 to +184 bp, respectively, were generated by PCR and cloned into the pGL3-basic luciferease reporter plasmid. The −1933 bp/+468-bp fragment was used as a template for PCR to generate a series of truncated *GFI1* promoter fragments. Single-stranded oligonucleotides corresponding to the different *GFI1* core promoter sequences of both strands were annealed *in vitro* and cloned into the *NheI* and *XhoI* sites of the pGL3-control luciferase reporter plasmid. Mutations in the *GFI1* promoter fragments were generated by site directed mutagenesis. HCT116 cells were transfected using *Trans*IT-LT1 Transfection Reagent and harvested 36 hrs later. Luciferase activities were measured using a Molecular Devices Lmax luminometer (Sunnyvale, CA) and normalized on the basis of the co-transfected β-galactosidase activity.

### Apoptosis Assay

Apoptosis was examined using the Annexin V-PE apoptosis detection kit (BD Biosciences). Briefly, 0.3×10^6^ cells were collected and incubated with Annexin V-PE and 7 amino-actinomycin (7-AAD). Cells were analyzed by two-color flow cytometry using BD Cellquest Pro software (Becton Dickinson, San Jose, CA).

### MTS Assay

Viable cell numbers were quantitated by the CellTiter 96® AQueous Non-Radioactive Cell Proliferation (MTS) Assay (Promega, Madison, WI). After extensive washing, 2×10^4^ cells were incubated in triplicate in 100 µl of RPMI 1640 medium in 96-well plates in the presence of Doxy (1 µg/ml) for 24 hrs, followed by treatment with doxorubicin (100 ng/ml for Ba/F3 cells and 2 mg/ml for Ramos cells) for 18 hrs. CellTiter 96® AQ_ueous_ One Solution Reagent was added to each well and the plates were read 2 hrs later at 490-nm wavelength in a microplate luminometer (Molecular Devices, Sunnyvale, CA).

### Statistics

GraphPad Prism software (GraphPad Software, La Jolla, CA, USA) was used for all statistical analysis. Data are shown as mean ± SD in all figures. A p value <0.05 was considered significant for all analyses and shown as *. ** denotes P<0.01, *** denotes P<0.001 and **** denotes p<0.0001. Significance of differences in cell viability was determined using Mann-Whitney U tests. Two-tailed paired T test was used to calculate differences of luciferase activities between samples in all luciferase assays, as well as differences of living cell number measured by trypan blue staining or MTS assays.

## Results

### p53 Inhibits the Expression of GFI1 Protein and mRNA

**Figure 1:**
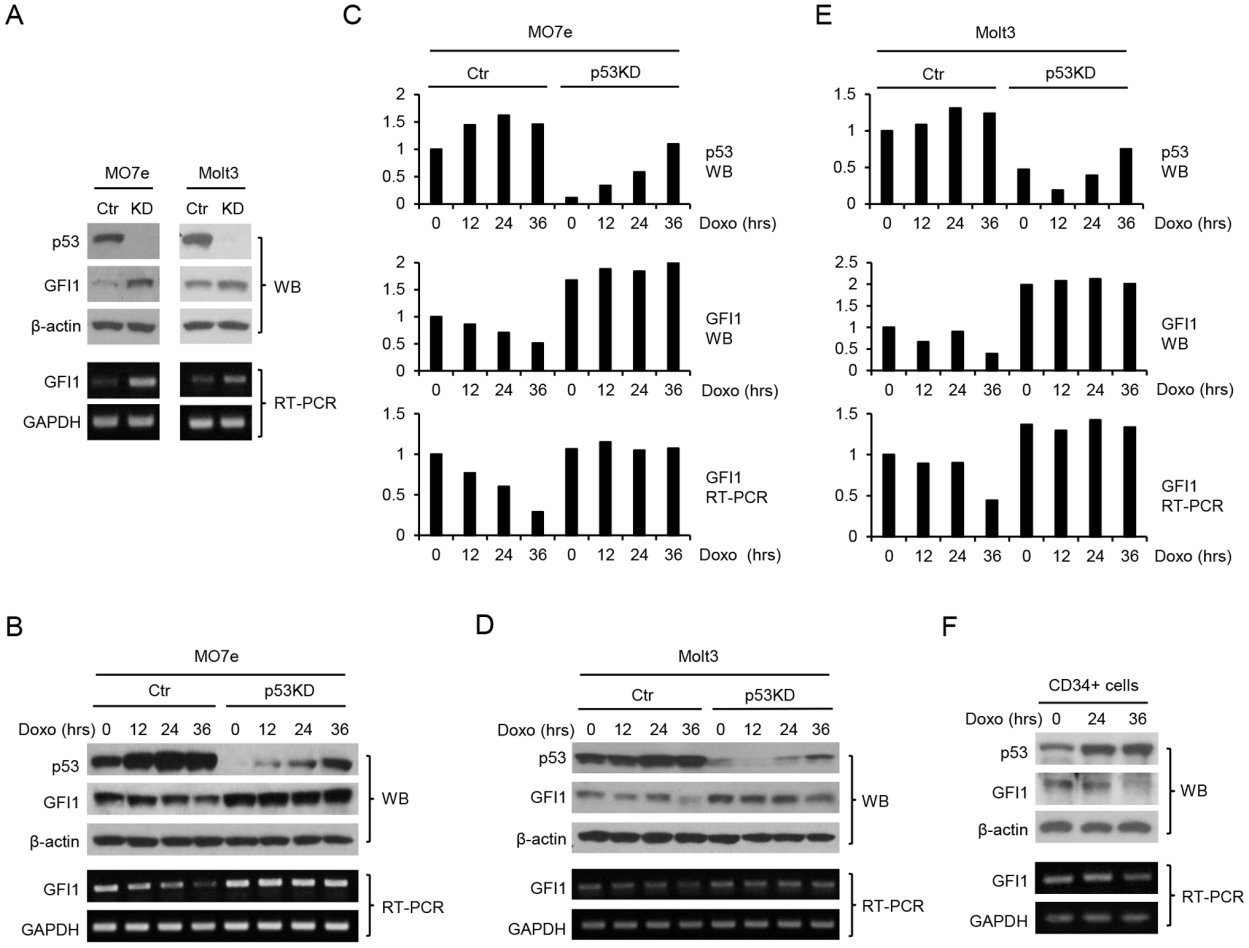
GFI1 protein and mRNA levels are upregulated upon p53 knockdown. (A) MO7e and Molt3 cells were infected with the lentivirus containing a p53 shRNA. The expression of GFI1 and p53 proteins was examined by Western blot analysis. GFI1 mRNA levels were examined by RT-PCR. Subsequently, control (Ctr) and p53 knocked down (KD) MO7e (B) and Molt3 (D) cells were treated with Doxo (25 ng/ml) for times as indicated and examined for expression of p53 and GFI1. The results shown in B and D were quantitated using ImageJ and normalized based on the levels of β-actin protein and GAPDH mRNA for MO7e (C) and Molt3 (E) cells. (F) Human umbilical cord blood CD34+ cells were treated with Doxo prior to evaluation of the expression of p53 and GFI1.

We explored the role of p53 in the regulation of GFI1 expression in hematopoietic cells. MO7e and Molt3 cells are human megakaryocytic and T lymphoblastic leukemia cell lines, respectively, that express wild type (WT) p53 protein. The expression of p53 in MO7e and Molt3 cells was knocked down through lentiviral mediated delivery of a p53 shRNA. Significantly, p53 knockdown was associated with increased expression of GFI1 at both protein and mRNA levels (Fig. 1A). Treatment with doxorubicin (Doxo), which induces topoisomerase II-mediated DNA double strand breaks, led to a steady increase in p53 protein level and this was accompanied by decreased GFI1 expression in both cell lines, which was most significant at 36 hrs of treatment (Figure 1B and C). However, GFI1 expression was not downregulated in the p53 knocked-down (KD) cells in which the levels of p53 remained low, albeit upregulated, following Doxo treatment. We also examined whether p53 upregulation correlated with GFI1 downregulation in human umbilical cord blood CD34^+^ cells. As shown in Fig. 1D, Doxo treatment upregulated p53 expression in the cells and this was associated with GFI1 downregulation at 36 hrs of treatment.

**Figure 2:**
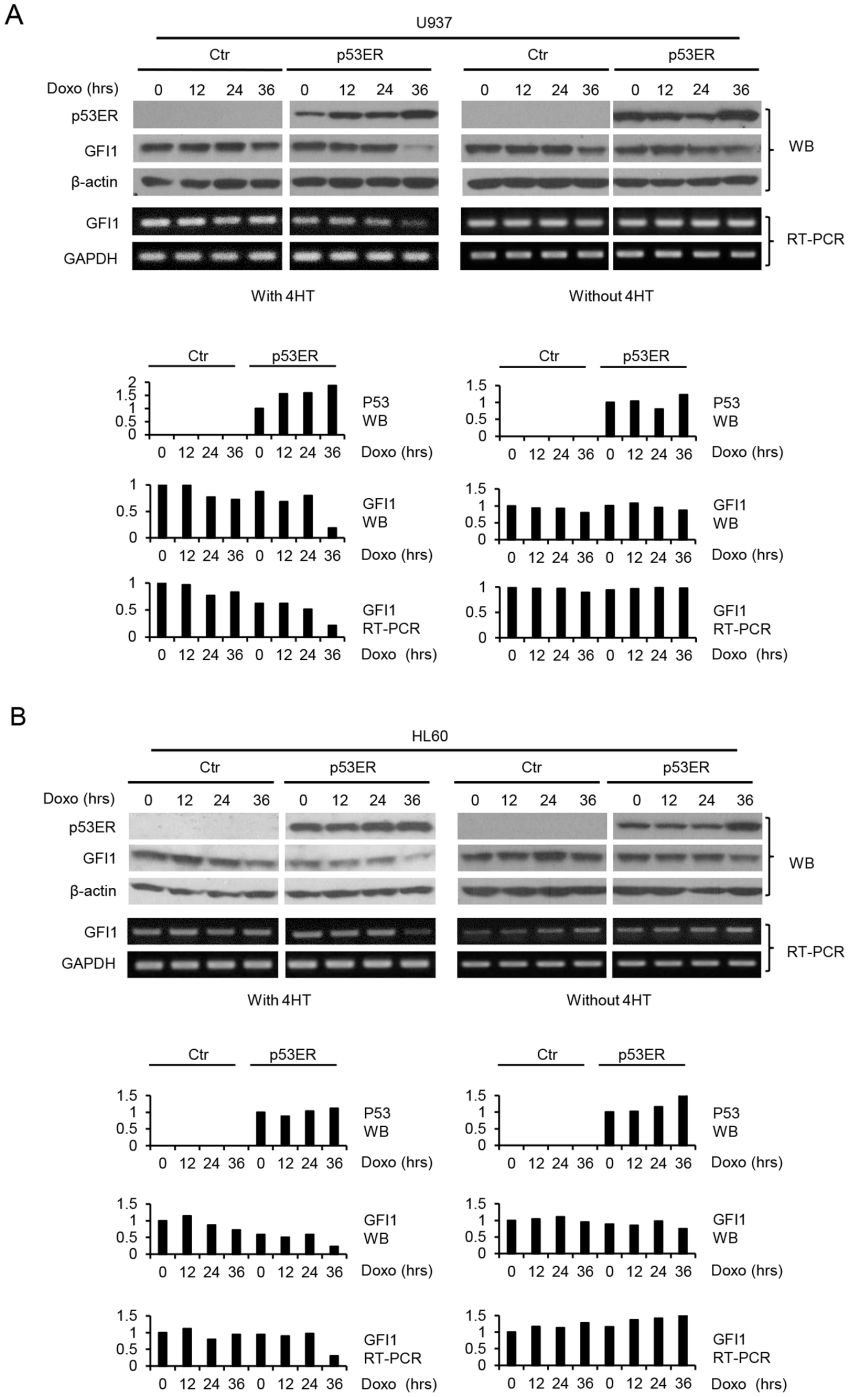
Restoration of p53 function results in GFI1 downregulation in response to DNA damage. U937 (A) and HL-60 (B) cells untransfected or transfected with p53ER^TAM^ were either left untreated or treated with Doxo (50 ng/ml) for times as indicated in the presence or absence of 4-HT. The expression of GFI1 and p53ER was examined by Western blotting and RT-PCR (upper panels), and the data were quantitated using ImageJ (lower panels). The moderate increase in GFI1 mRNA level following Doxo treatment in the control HL-60 cells without Doxo treatment was not reproducible in two other independent experiments.

We further assessed the effect of restoration of p53 function on GFI1 expression in *p53*-deficient cells. Human myeloid leukemic U937 and HL-60 cells, which lacked functional p53, were transduced with the retroviral expression construct for the p53/estrogen receptor ligand binding domain fusion protein (p53ER^TAM^). It is worth noting that addition of 4-hydroxy tamaxifen (4-HT) does not overly activate p53ER^TAM^, but enables p53ER^TAM^ to be activated by upstream signals [38] (data not shown). Cells were subsequently treated with Doxo in the absence or presence of 4-HT. As shown in Fig. 2, GFI1 protein and mRNA levels in the p53ER^TAM^-expressing cells declined significantly at 36 hrs of Doxo treatment in the presence of 4-HT, but did not or only slightly declined in its absence. GFI1 expression was not or barely downregulated by Doxo treatment in the control cells with or without added 4-HT. Together, these data demonstrated that p53 inhibited GFI1 expression, presumably by targeting its mRNA.

### p53 Represses *GFI1* Transcription through Direct Binding to its Promoter

**Figure 3:**
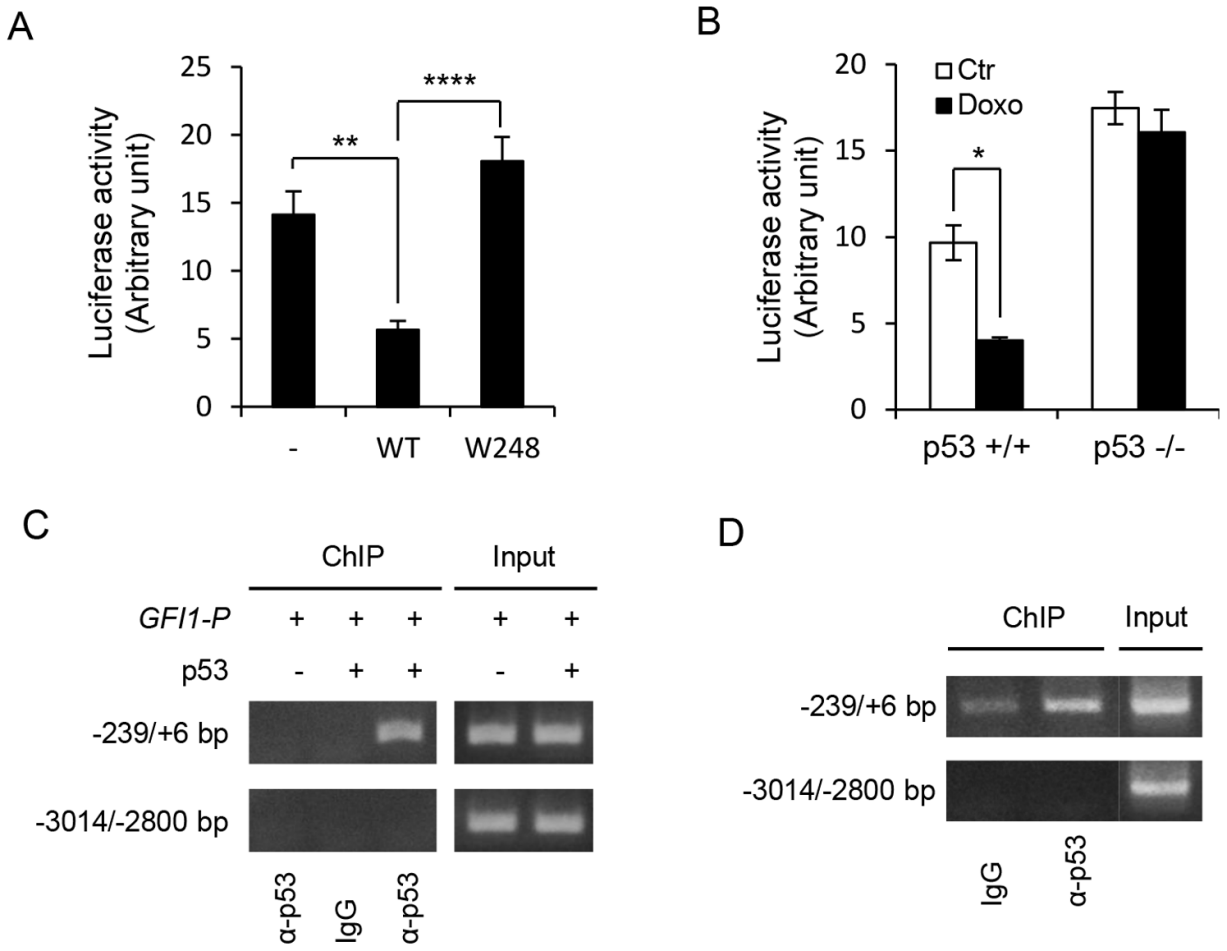
p53 binds to and represses *GFI1*. (A) *p53* ^−/−^ HCT116 cells were transfected with the *GFI1* promoter (−1933/+468 bp) luciferase reporter construct along with the wild type (WT) or W248 mutant p53. Luciferase activities were measured 36 hrs later and normalized for β-Gal activities. Data are shown as mean ± SD. (B) *p53* ^+/+^ and *p53* ^−/−^ HCT116 cells were transfected with *GFI1* promoter luciferase reporter construct and treated with Doxo (400 ng/ml) 8 hrs later. Luciferase activities were measured 16 hrs after Doxo treatment. (C) *p53* ^−/−^ HCT116 cells were transfected with pGL3-basic plasmid containing *GFI1* promoter fragment (−4840/+184 bp) either alone or together with p53. ChIP assays were carried out using the anti human p53 or an irrelevant species-matched antibody. The indicated regions of *GFI1* promoter were amplified by PCR. (D) ChIP experiment was carried out on Molt3 cells using the anti human p53 and control antibodies.

We examined whether p53 repressed the activity of a human *GFI1* promoter fragment spanning from −1933 bp to +468 bp (relative to human *GFI1* mRNA sequence [NM_005263]) in the *p53* ^−/−^ HCT116 cells. As shown in Fig. 3A, luciferase activity driven by the *GFI1* promoter was markedly inhibited by the wild type (WT) p53, but not by the W248 mutant which is defective in DNA binding, indicating that DNA binding activity was required for p53 repression of *GFI1* promoter. Consistent with the role of p53 in repressing *GFI1*, Doxo treatment inhibited the activity of the *GFI1* promoter fragment in the *p53* ^+/+^ HCT116 cells, but not in the *p53* ^−/−^ cells (Fig. 3B).

We further performed ChIP assays to investigate whether p53 bound to the *GFI1* promoter. The *p53* ^−/−^ HCT116 cells were transiently transfected with a human *GFI1* promoter fragment spanning from −4840 bp to +184 bp either alone or together with WT p53 expression construct. Protein-DNA complexes were prepared and immunoprecipitated with an anti-human p53 antibody or a species-matched control antibody. As shown in Figure 3C, p53 specifically bound to the proximal, but not the distal, promoter region of *GFI1*. ChIP assays were also carried out on Molt3 cells to address whether p53 bound to *GFI1* in the endogenous setting. As in the *p53* ^−/−^ HCT116 cells, endogenous p53 bound to the *GFI1* core promoter region (Fig. 3D). Together, these data indicate that p53 repressed *GFI1* transcription by directly binding to its promoter.

### The *GFI1* Core Promoter Contains a p53 RE

**Figure 4:**
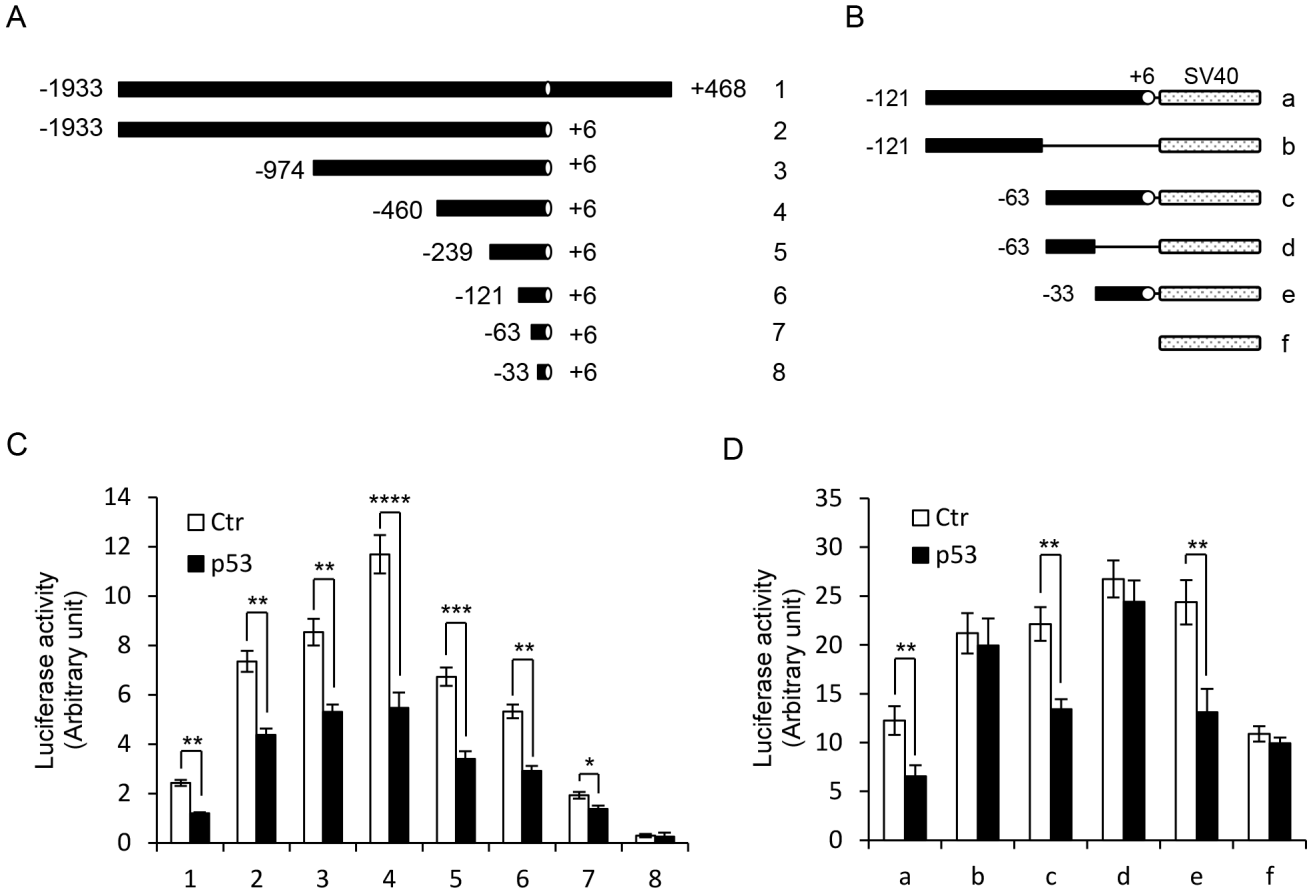
Mapping of the *GFI1* promoter region required for p53-mediated repression. (A and B) Schematic diagrams of *GFI1* promoter fragments cloned into pGL3-basic vector (A) or inserted upstream of the *SV40* promoter of the pGL3-promoter vector. (C and D) p53^−/−^ HCT116 cells were transfected with the indicated *GFI1* promoter luciferase reporter constructs without or with p53. Luciferase activities were measured 36 hrs after transfection.

To identify the p53 RE in the *GFI1* promoter, we assessed the effects of p53 on the activities of a series of progressively truncated *GFI1* promoter fragments (Fig. 4A). Notably, the promoter fragment spanning from −63 bp to +6 bp was still repressed by p53, but further truncation resulted in loss of promoter activity (Fig. 4C). To precisely define the *GFI1* core promoter region required for p53-mediated repression, we determined which *GFI1* core promoter region was sufficient to confer p53 response to a non-p53 responsive promoter. A number of oligonucleotides corresponding to the different regions of *GFI1* core promoter were synthesized and inserted upstream of the *SV40* promoter of the pGL3 control plasmid (Fig. 4B). The *SV40* promoter was not repressed by p53 (Fig. 4D). Insertion of any of the three oligonucleotides containing the 39-bp core promoter sequence (−33 bp/+6 bp) resulted in the repression of the *SV40* promoter by p53. In contrast, the two oligonucleotides lacking this sequence had no effect on p53 response. These data demonstrated that the p53 RE is located within the *GFI1* core promoter region between −33 bp and +6 bp.

**Figure 5:**
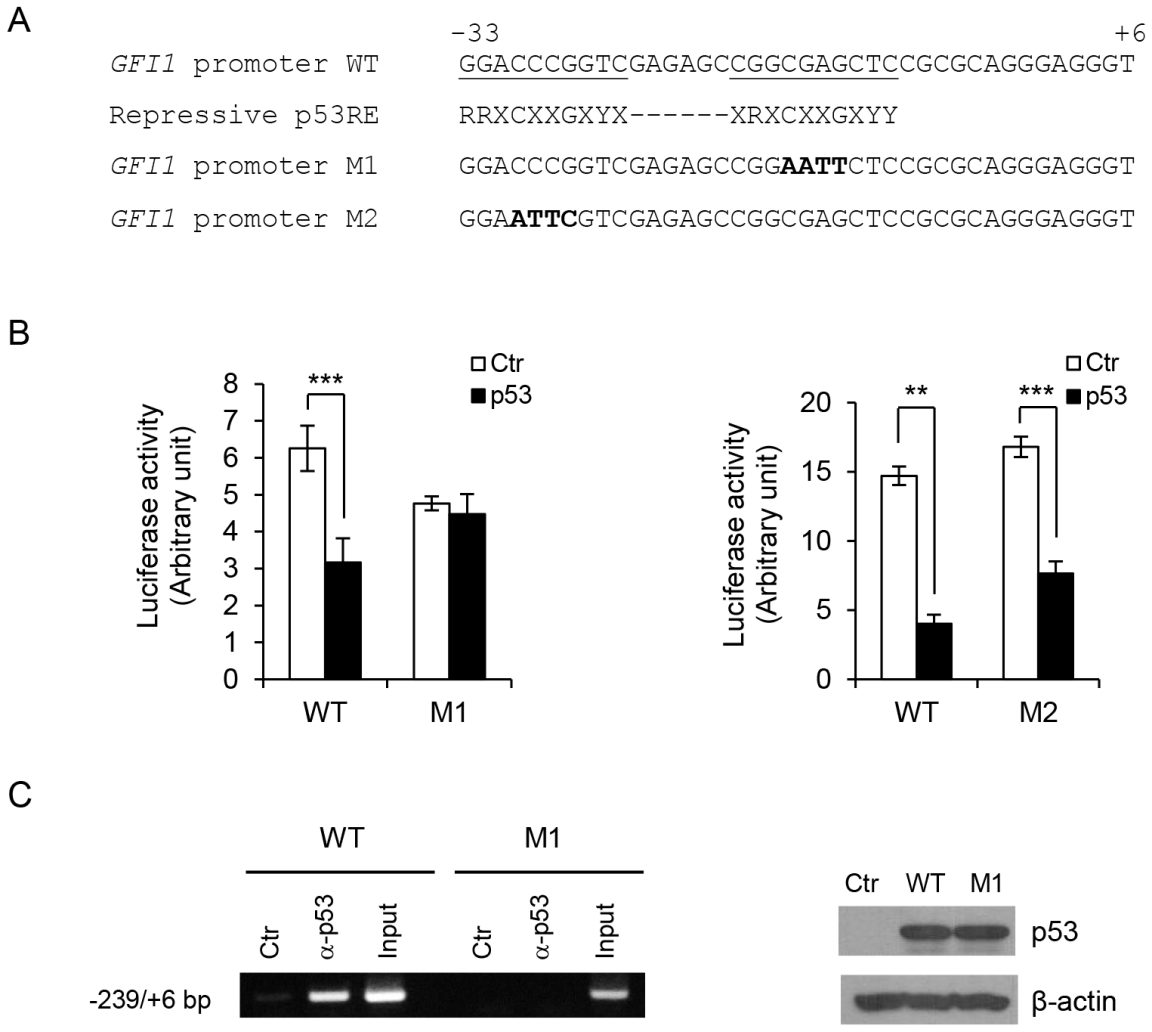
Identification of the repressive p53 RE in *GFI1* core promoter. (A) Nucleotide sequences of wild type (WT) and mutated *GFI1* core promoter fragments as compared with conserved repressive p53 RE. Potential p53 RE in *GFI1* core promoter are underlined. Mutated nucleotides are in bold. (B) *p53* ^−/−^ HCT116 cells were transfected with WT or M1 *GFI1* promoter (−1933/+468 bp; left panel), or WT or M2 *GFI1* promoter (−460/+6 bp; right panel) luciferase reporter construct without or with p53. Luciferase activity was measured 36 hrs after transfection. (C) *p53* ^−/−^ HCT116 cells were transfected with pGL3-basic plasmid containing WT or M1 *GFI1* promoter fragment (−4840/+184 bp) along with p53. Binding of p53 to the *GFI1* promoter fragments was examined by ChIP assays (left panel). Expression of p53 was confirmed by Western blot analysis (right panels).

A close examination of *GFI1* core promoter sequence revealed that the promoter region spanning from −34 bp to −9 bp appeared to match the loosely defined repressive p53 RE [39]. We therefore addressed whether mutation of the two half-sites in the potential repressive p53 RE of *GFI1* promoter blocked p53-mediated repression. Interestingly, mutation of the CGAG sequence in the downstream half-site, but not the CCCG sequence in the upstream half-site, abolished p53 repression of the *GFI1* promoter (Fig. 5B). Consistent with loss of repression by p53, ChIP assays demonstrated that p53 was unable to bind to the *GFI1* promoter when the downstream half-site was mutated (Fig. 5C). Together, these data indicated that the *GFI1* core promoter contains a p53 RE that is required for p53-mediated repression.

### Repression of *GFI1* by p53 is not Dependent on Histone Deacetylases (HDACs) and p21^Cip1^

**Figure 6:**
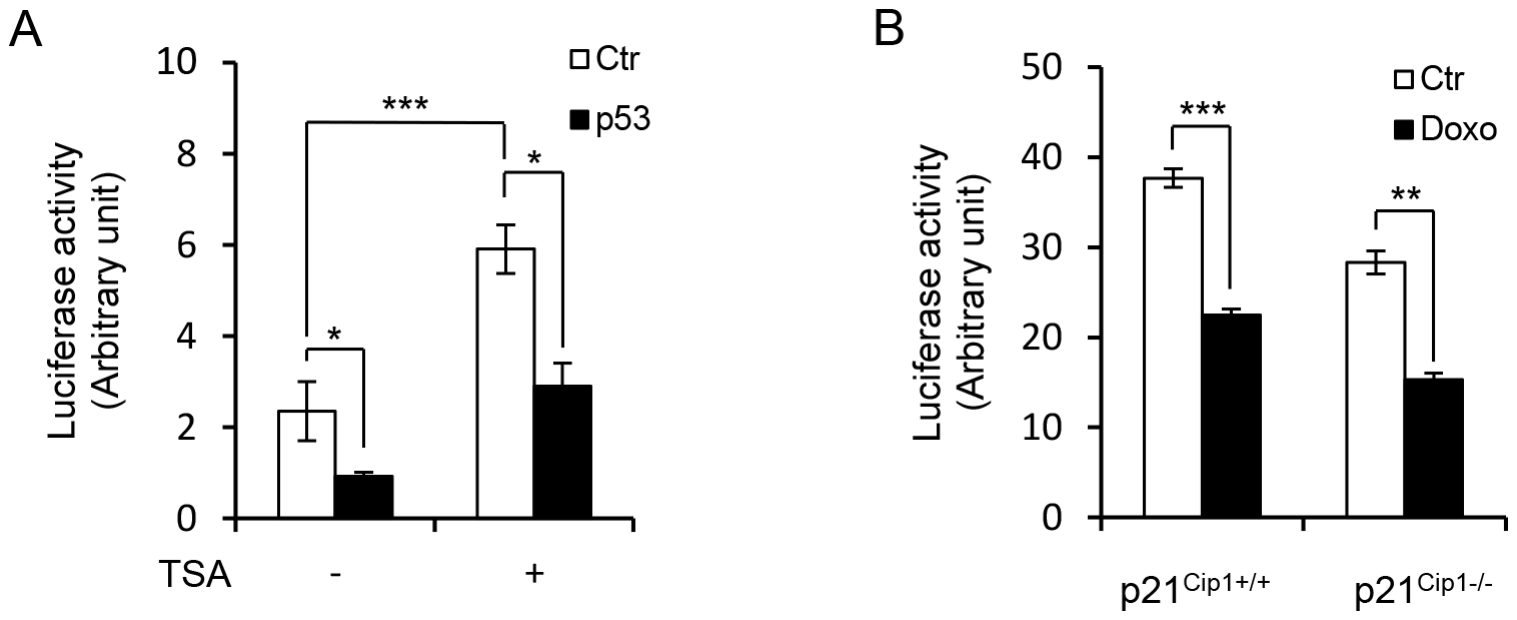
Repression of *GFI1* by p53 is independent of HDACs and p21^Cip1^. (A) *p53* ^−/−^ HCT116 cells were transfected with *GFI1* promoter (−1933/+468 bp) luciferase reporter construct without or with p53 and treated with TSA (0.15 µM) 8 hrs later. Luciferase activities were measured 24 hrs after TSA treatment. (B) *p21^Cip1^* ^+/+^ and *p21^Cip1^* ^−/−^ HCT116 cells were transfected with *GFI1* promoter (−1933/+468 bp) reporter construct and treated with Doxo (400 ng/ml) 8 hrs later. Luciferase activities were measured 16 hrs after Doxo treatment.

Recent studies have indicated that p53 may repress certain target genes through the recruitment of HDACs via its interaction with mSIN3a [26], [40]. We therefore addressed whether trichostatin A (TSA), a potent HDAC inhibitor, would affect p53-mediated repression of *GFI1*. TSA treatment significantly augmented the activity of the *GFI1* promoter, but did not abolish p53-mediated repression (Fig. 6A), suggesting that HDAC function is involved in the negative regulation of *GFI1* transcription, but is not required for p53-mediated repression. Consistent with this, forced expression of p53 in *p53* ^−/−^ HCT116 cells did not enhance mSIN3A binding to the *GFI1* promoter (data not shown).

p53 has also been shown to repress its target genes indirectly through transcriptional activation of *p21^Cip1^*, which inhibits the phosphorylation of retinoblastoma protein, leading to decreased activation of E2F-regulated genes [26], [40]. To exclude this possibility, we examined whether Doxo was able to inhibit *GFI1* promoter activity in *p21^Cip1−/−^* HCT116 cells. As shown in Fig. 6B, the activity of the *GFI1* promoter was suppressed upon Doxo treatment in both *p21^Cip1+/+^* and *p21^Cip1−/−^* HCT116 cells, indicating that repression of *GFI1* by p53 is independent of p21^Cip1^. In support of this, luciferase reporter assays indicated that E2F1 was unable to activate the *GFI1* promoter (data not shown).

### Gfi1 Inhibits DNA Damage Induced Apoptosis in a p53-independent Manner

**Figure 7:**
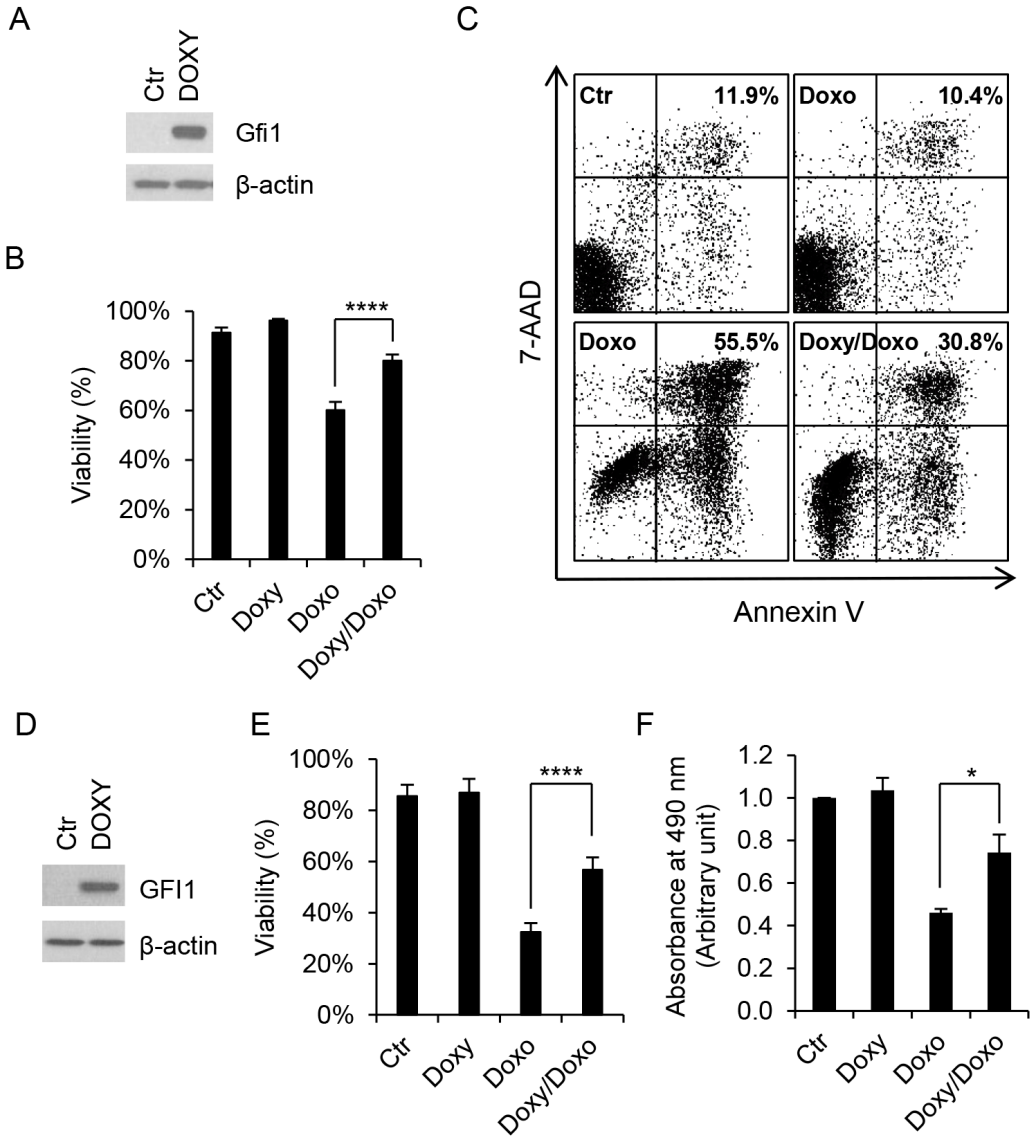
Overexpression of Gfi1 inhibits DNA damage-induced cell death. Ba/F3 (A–C) and Ramos (D–F) cells were transduced with the inducible lentiviral expression construct for Gfi1 and examined for Gfi1 expression by Western blot analysis after incubation with Doxy (1 µg/ml) for 24 hrs (A and D). Cells were then exposed to Doxo (100 ng/ml for Ba/F3 cells and 2 mg/ml for Ramos cells) for 18 hrs with or without preincubation with Doxy (1 µg/ml) for 24 hrs. Cell viabilities were determined by exclusion of trypan blue staining (B and E), percentages of apoptotic (annexin V-positive) cells were assessed by flow cytometry after staining with annexin V and 7-AAD (C), and the numbers of living cells were quantitated by MTS assay (F).

We addressed the role of GFI1 in DNA damage-induced apoptosis. Murine pro-B Ba/F3 cells were transduced with a lentiviral construct that expressed Gfi1 from the tetracycline-response element (TRE) in the presence of Doxy. The expression of endogenous Gfi1 was barely detectable in Ba/F3 cells by Western blot analysis, but was strongly induced upon addition of Doxy (Fig. 7A). Cells were then treated with Doxo and evaluated for apoptotic cell death by trypan blue staining and annexin V assays. Treatment of Ba/F3 cells with Doxo led to markedly reduced cell viability and increased number of cells undergoing apoptosis (Fig. 7B and C). Notably, Doxo-induced apoptosis was significantly reduced following addition of Doxy.

To address whether the protective effect of Gfi1 on cell death was dependent on functional p53, we introduced the Doxy-responsive Gfi1 lentiviral construct into Ramos cells (Fig. 7D), a *p53*-deficient Burkitt lymphoma cell line. Ramos cells were highly resistant to DNA damage, but treatment with Doxo at 2 µg/ml resulted in a significant decrease in cell viability and the number of living cells as determined by exclusion of trypan blue staining and MTS assay (Fig. 7E and F). MTS assay instead of annexin V staining was used because Doxo at 2 µg/ml interfered with flow cytometric analysis of annexin V stained cells (data not shown). As in Ba/F3 cells, Doxo-induced cell death was significantly inhibited upon Gfi1 induction in Ramos cells, indicating that Gfi1 inhibition of Doxo-induced cell death was independent of p53.

**Figure 8:**
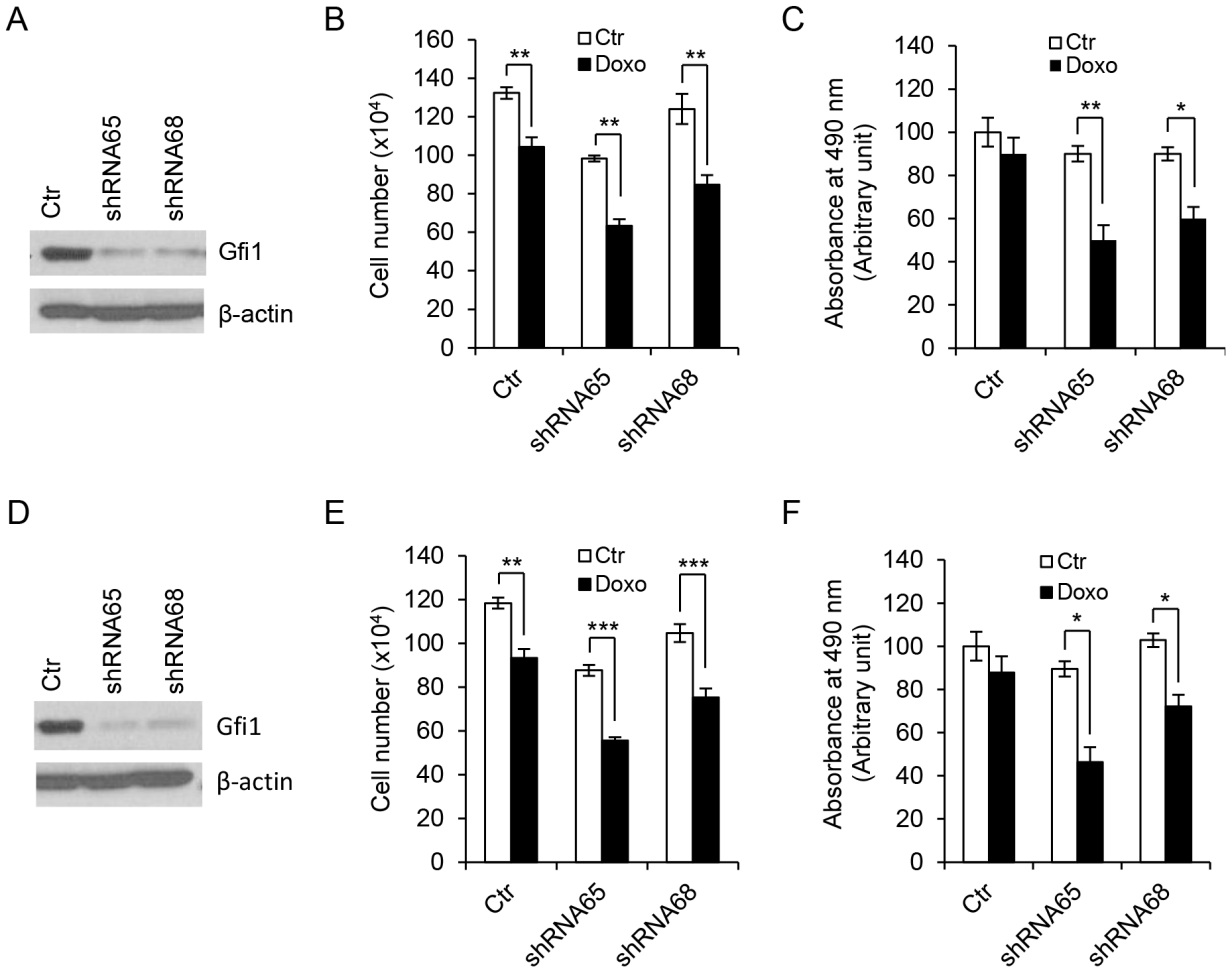
Knockdown of GFI1 increases cell death in response to DNA damage. U937 (A–C) and HL-60 (D–F) cells were transduced with empty lentivirus (Ctr) or lentiviruses containing two different shRNAs against *GFI1* and examined for GFI1 expression by Western blot analysis (A and D). Cells were left untreated or treated with Doxo at 200 ng/ml for 10 hrs prior to evaluation of living cell numbers by trypan blue exclusion (B and E) and MTS assays (C and F).

We further assessed the effect of GFI1 knockdown on Doxo-induced cell death in myeloid leukemia cell lines U937 and HL-60, both of which expressed high levels of endogenous GFI1 [36], [37]. GFI1 expression was knocked down in cells transduced with the lentiviral vectors containing two different GFI1 shRNAs, but not with the empty vector (Fig. 8). Notably, GFI1 knockdown was associated with a significant increase in the sensitivity of cells to Doxo-induced cell death. Because U937 and HL-60 cells lacked functional p53, these data further indicate that the protective effect of GFI1 on DNA damage-induced apoptosis was independent of p53 in hematopoietic cells.

### GFI1 does not Affect the Expression of Bax, Bak and Bcl-2

**Figure 9:**
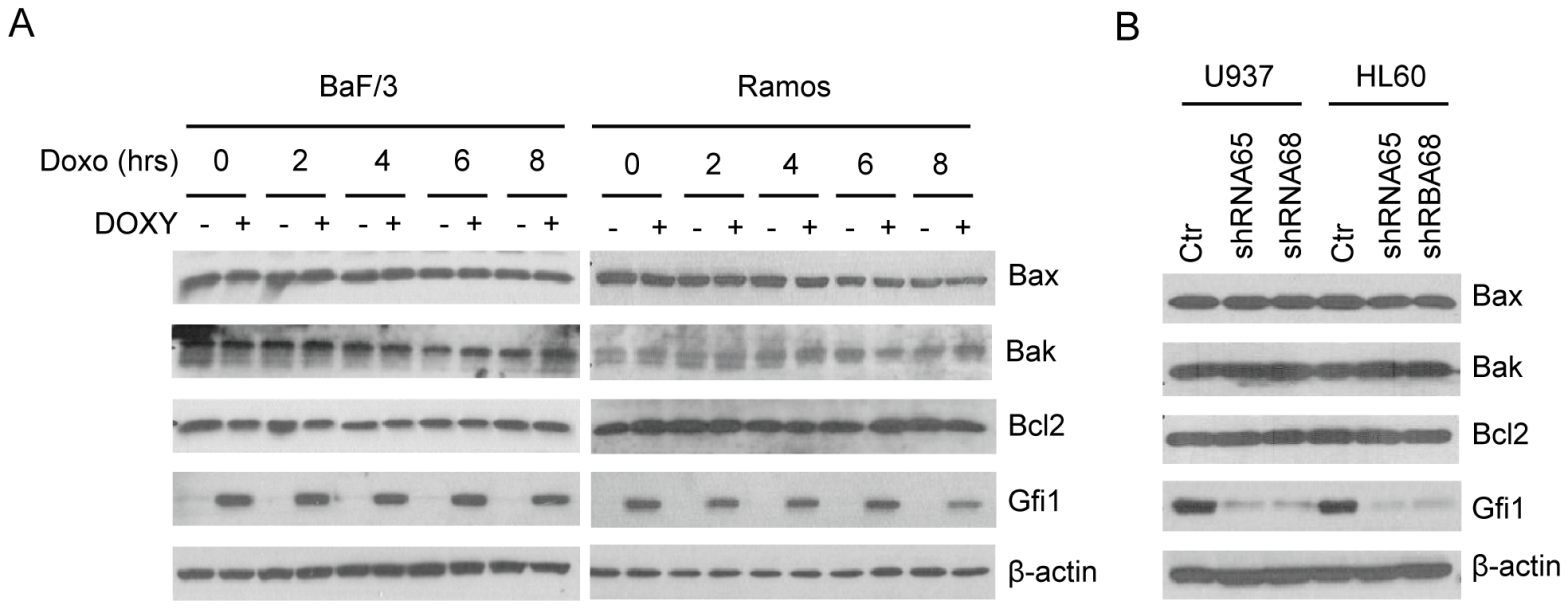
Gfi1 has no significant effect on the expression of Bax, Bak and Bcl-2. (A) Ba/F3 and Ramos cells transduced with the inducible Gfi1-expressing lentiviral construct were preincubated with Doxy (1 µg/ml) for 24 hrs and then treated with Doxo (100 ng/ml) for times as indicated. The expression of Gfi1 and the Bcl-2 family members as indicated was examined by Western blot analysis. (B) The levels of the Bcl-2 family members in control and GFI1 knocked down U937 and HL-60 cells were examined by Western blot analysis.

Gfi1 has been shown to repress the pro-apoptotic Bcl-2 family members *Bax* and probably also *Bak* [16], [41], but appears to upregulate the expression of the anti-apoptotic Bcl-2 in hematopoietic cells [5], [42]. To investigate the mechanism whereby Gfi1 inhibited DNA damage-induced apoptosis, we examined the expression of Bax, Bak and Bcl-2 in Ba/F3 and Ramos cells transduced with the Doxy-responsive Gfi1 expression construct as well as in the GFI1 knocked down HL-60 and U937 cells. As shown in Fig. 9, the expression of these Bcl-2 family members was not affected by Gfi1 induction or GFI1 knockdown. Treatment of Ba/F3 and Ramos cells with Doxo following Gfi1 induction also had no apparent effects on the expression of these Bcl-2 family members. Thus, Bax, Bak and Bcl-2 are unlikely the major players in Gfi1-mediated inhibition of DNA damage-induced apoptosis in the cell lines used in this study.

## Discussion

In this paper, we have demonstrated that *GFI1* transcription is repressed by p53 in hematopoietic cells. We have shown that p53 knockdown is associated with increased expression of GFI1 at both mRNA and protein levels, and abolishes GFI1 downregulation in response to DNA damage. In contrast, restoration of p53 function in *p53*-deficient cells results in reduced GFI1 expression and rescues DNA damage-induced GFI1 downregulation. We have also shown that p53 binds to and represses the *GFI1* core promoter, and that the DNA binding activity of p53 is indispensable for its repression of *GFI1*. Consistent with this, the *GFI1* core promoter contains a p53 RE that is required for p53 binding and repression. Together, these data establish for the first time that p53 represses *GFI1* transcription by directly binding to its core promoter and add *GFI1* to the increasing list of genes that are repressed by p53. Our data have important implications for understanding the role of GFI1 in normal hematopoiesis and lymphoid malignancies.

In addition to transcriptional activation, p53 has been shown to repress a number of genes and recent studies indicate that transcriptional repression by p53 is required for its tumor suppressor function [24], [26], [39], [40]. Mutations in *TP53* encoding p53, while not as prevalent as in solid tumors, have been identified in approximately 20–30% of patients with lymphoid malignancies including lymphomas and appear to be a prognostic indicator predicting inferior survival [43], [44]. Gfi1 has been shown to function as an oncoprotein in lymphoid cells and collaborate with c-Myc in lymphomagenesis. We recently demonstrated that Gfi1 forms a ternary complex with c-Myc through interaction with Miz-1, and Gfi1 and c-Myc act in collaboration to repress *p15^INK4B^* and *p21^Cip1^* [36], [37], the latter being a p53-activated target gene. Interestingly, like *GFI1*, *c-Myc* is also repressed by p53 in hematopoietic cells [45]. Therefore, it is likely that p53 suppresses lymphomagenesis in part through repression of *Gfi1* and *c-Myc*.

An important mechanism by which p53 regulates gene expression is through direct binding to the REs in the promoters of its target genes. The canonical p53 RE involved in p53-mediated transcriptional activation has been defined as two 10-bp half-sites 5′-RRRCWWGYYY-3′ separated by a spacer of 0–13 bp (R is purine, Y is pyrimidine and W is A or T) with certain level of tolerance to mismatches from the consensus sequence [26], [39], [40]. Significantly less is known about the p53 REs involved in p53-mediated repression and a number of models of repressive p53 REs have been proposed. Our data indicate that the 39-bp *GFI1* core promoter is sufficient for p53 binding to and repression of *GFI1*. Interestingly, this core promoter region appears to contain the repressive p53 RE loosely defined as RRXCXXGXYX-XRXCXXGXYY (X can be A, C, G or T) [39]. However, mutation of the CGAG sequence in the downstream half-site, but not the CCCG sequence in the upstream half-site, abolishes p53 binding to and repression of *GFI1*. Thus, the precise sequence or motif of the repressive p53 RE in the *GFI1* core promoter remains to be determined. Irrespectively, our data indicate that the 39-bp core promoter of *GFI1* contains a p53 RE that is required for p53-mediated repression.

It is still unclear how p53 represses *GFI1* transcription through the repressive p53 RE. Although p53 has been shown to recruit HDACs to its target genes such as *c-Myc*, *survivin*, *MAP4, Nanog* and *Arf* [24], [26], [39], [40], [46], our data indicate that HDAC function is not required for p53-mediated repression of *GFI1*. Additionally, our data are in sharp contrast to a previous study showing that p53 binds to and activates mouse *Gfi1* [47]. In this study two putative p53 REs were identified in the mouse *Gfi1* promoter, but only RE1 is required for p53-mediated activation of a ∼0.75-kb mouse *Gfi1* promoter fragment in human Hela cells. The reason for the discrepancies is unknown.

We have further demonstrated that GFI1 acts to inhibit cell death induced by DNA damage, consistent with recent reports that *Gfi1* ^−/−^ HSCs and lymphoblastic leukemia cells exhibited increased rates of apoptosis in response to DNA damage [5], [19]. Thus, it is possible that upon activation by DNA damage, p53 may induce apoptosis in part through repression of *GFI1*. Interestingly, Gfi1 has been shown to restrict p53-dependent DNA damage response, which may be responsible for its anti-apoptotic activity [19]. However, our data suggest that the protective effect of Gfi1 is not dependent on p53 as Gfi1 also protects cells lacking functional p53 from DNA damage-induced cell death. Gfi1 has been shown to repress the pro-apoptotic Bcl-2 family members *Bax* and probably also *Bak* [16], [41]. In line with these studies, Bax expression was significantly augmented following irradiation in *Gfi1* ^−/−^ HSCs and thymocytes [5], [19]. In contrast, the expression of the pro-survival Bcl-2 appeared to be downregulated in *Gfi1* ^−/−^ T cells and HSCs [5], [42]. However, we failed to observe any significant effects of GFI1 overexpression or knockdown on the expression of these Bcl-2 family members in the hematopoietic cell lines used in this study. Further studies are needed to elucidate the molecular mechanism by which GFI1 inhibits DNA damage-induced apoptosis.
